# Public Health Risk of Foodborne Pathogens in Edible African Land Snails, Cameroon 

**DOI:** 10.3201/eid2808.220722

**Published:** 2022-08

**Authors:** Mary Nkongho Tanyitiku, Graeme Nicholas, Igor C. Njombissie Petcheu, Jon J. Sullivan, Stephen L.W. On

**Affiliations:** Lincoln University, Christchurch, New Zealand (M.N. Tanyitiku, G. Nicholas, I.C. Njombissie Petcheu, J.J. Sullivan, S.L.W. On);; Global Mapping and Environmental Monitoring, Yaounde, Cameroon (I.C. Njombissie Petcheu).

**Keywords:** African land snails, natural habitats, foodborne pathogens, potential health risks, Cameroon, Campylobacter spp., Yersinia spp., Listeria spp., Salmonella spp., Shiga toxin–producing Escherichia coli, STEC, food safety, bacteria, enteric infections

## Abstract

In tropical countries, land snails are an important food source; however, foodborne disease risks are poorly quantified. We detected *Campylobacter* spp*., Yersinia* spp., *Listeria* spp., *Salmonella* spp., or Shiga-toxigenic *Escherichia coli* in 57%–86% of snails in Cameroon. Snail meat is a likely vector for enteric diseases in sub-Saharan Africa countries.

African land snails (*Achatina achatina, Achatina fulica, Archachatina marginata*) are a source of food for many persons in sub-Saharan Africa (*1*–*5*). Snail meat contains 37%–51% protein, which is higher than the protein content in poultry (18.3%), fish (18.0%), cattle (17.5%), sheep (16.4%), and swine (14.5%) (*1*,*2*,*5*).

In rural settings, commercial snail farming is uncommon. Rural dwellers may spend up to 20 hours a week in search of edible snails in environments that include marshes, decaying vegetation, domestic wastes, roadsides, footpaths, and bushes (*2*,*4*–*6*). Those local practices of collecting, handling, and consuming snails could expose handlers and consumers to foodborne pathogens.

Although several studies (*2*,*3*,*6*) have highlighted the close association of edible snails with pathogenic microorganisms, their potential contribution to the burden of foodborne diseases in Africa has been overlooked. In Cameroon, no data on foodborne pathogens in snail meat are available, and their role in causing enteric diseases in the local population is unknown. Our study assessed the prevalence of potential foodborne pathogens in African land snails consumed in Buea, Cameroon.

We collected live snails from 3 locations (in persons’ homes, on arable land, and in local markets) during June–October 2019. We sampled within persons’ homes from 9 PM to 5 AM on rainy nights and on arable land during the day. In Buea, live snails are found actively moving around at night, and during the day, they usually are present underneath decaying vegetation in farmlands (*7*). We purchased samples from local markets weekly from snail vendors. Our choice of these sampling locations emerged from participants’ responses to questions such as, “Where do you get the snails you eat or sell at the market?”; “How do you get the snails you eat or sell?”; “How do you know snails are present there?”; and “If you are to teach your daughter on how to get snails, what will you teach her?” (*7*)

We collected live snails weekly from the 3 locations and stored them at room temperature in a laboratory in 2-L sterile Sistema containers (Sistema Plastics, https://www.sistemaplastics.com). We aseptically collected the feces of 6–12 edible snails/sample within 12–18 hours, pooled them, and placed them in 15-mL sterile tubes manufactured by Eppendorf (https://corporate.eppendorf.com). We then stored the samples at –80°C before DNA extraction. We then stored DNA extracts at 4°C before air freighting them to Lincoln University (Christchurch, New Zealand), for PCR analysis. We examined for the presence of Shiga toxin–producing *Escherichia coli*, *Campylobacter* spp., *Salmonella* spp., *Listeria* spp., and *Yersinia* spp. by using a high-fidelity DNA polymerase (repliQa Hifi toughmix; Quantabio, https://www.quantabio.com) (Appendix). We validated PCR methods in-house by using authenticated reference strains as positive and negative controls and then detecting them by electrophoresis. We recorded the presence of an amplicon of the appropriate size for each PCR in each sample as a positive result. For Shiga toxin–producing *Escherichia coli*, a positive result required the detection of both *stx1* and *stx2* genes. These criteria determined the occurrences of each pathogen in the samples (Table; Figure).

**Table:** Frequency of pathogens detected by PCR in African land snails, Buea, Cameroon, June–October 2019*

Pathogen	STEC	*Campylobacter* spp.	*Salmonella* spp.	*Listeria* spp.	*Yersinia* spp.
Frequency, %	57	75	69	86	71

*STEC, Shiga toxin–producing *Escherichia coli.*

**Figure:**
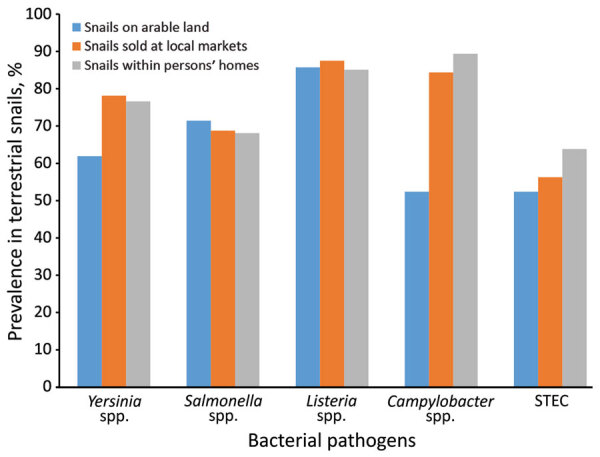
Prevalence of foodborne pathogens in land snails sampled in 3 selected locations, Buea, Cameroon. June–October 2019. STEC, Shiga toxin–producing *Escherichia coli.*

We detected >1 pathogen in every sample examined; most samples contained multiple pathogens. We also calculated the prevalence of each pathogen within the 3 sampling locations (Figure). The overall pathogen prevalence among the samples examined was high, ranging from 57% to 86%.

Although detailed information regarding the consumption of snail meat is not available in Cameroon, live snails are sold in almost every local market in the country (*8*). As for other sub-Saharan countries, an increase in the demand for snail meat has prompted the random collection of edible snails from locations that could be considered unhygienic (*2*,*3*,*6*)*.* Our results identify the public health risks in the handling and consumption of raw or undercooked edible snails collected from natural habitats in Cameroon. Similar pathogenic microorganisms have been isolated in edible snails consumed in Nigeria (*2*) and Ghana (*3*,*6*).

Moreover, the pathogens isolated in this study are associated with many foodborne outbreaks in developed countries such as the United States (*9*). Higher prevalences of *Campylobacter* spp. (75.37%) and *Listeria* spp. (86.10%) may reflect the common practice of free-range poultry farming in Buea and the direct contact of snails with the soil and decaying vegetation (*3*,*6*). Although previous studies highlighted that the local residents believed their practices of snail washing with aluminum sulfate or salt and lime in addition to boiling and then stewing could kill all microorganisms (*3*,*7*), Akpomie et al. (*2*) described substantial bacterial loads in snail meat after boiling, frying, smoking, and oven drying in Nigeria. Thus, our results strongly suggest that foodborne outbreaks from edible snail consumption may be occurring, but are unidentified, in Cameroon, and probably other sub-Saharan Africa countries. The situation clearly indicates a pressing need for interventions to improve public health, for which best results may be obtained in conjunction with a deeper understanding of community attitudes and practices (*7*,*10*).

Appendix Additional information about public health risk of foodborne pathogens in edible African land snails, Cameroon.
