# Supplementary material for: Public Health Risk of Foodborne Pathogens in Edible African Land Snails, Cameroon
Source: Emerg Infect Dis. 2022 Aug;28(8):1715–7. doi: 10.3201/eid2808.220722 (PMC9328896; doi:10.3201/eid2808.220722)
Supplement: Appendix — Additional information about public health risk of foodborne pathogens in edible African land snails, Cameroon. [file 22-0722-Techapp-s1.pdf]

Article DOI: https://doi.org/10.3201/eid2808.220722

Public Health Risk of Foodborne Pathogens in Edible African Land Snails, Cameroon

Appendix

Detailed Methods

DNA Extraction

The manufacturers guidelines of the Presto stool gDNA extraction kit were followed. 200mg of snail feces was centrifuged at 8000 g for 2mins in 800µl ST1 buffer solution and incubated at 70°C for 5mins. 500µl of supernatant was placed in a 1.7ml microcentrifuge tube containing 150µl of ST2 buffer, briefly vortex, and incubated at±4°C for 5 min. The mixture was centrifuged at 16000 g for 3 minutes and a clear supernatant of 500µl of was transferred to the inhibitor removal column. It was then centrifuged at 16000 g for 1 min and the column was discarded. 800µl of ST3 buffer was added to the flow through and then to a new GD column and centrifuged at 1600 g for 30 sec. This process was repeated three times to completely wash the bounded DNA. 100µl of preheated 10 mM Tris-HCl, 1mM EDTA, pH8.0 was added at the center of the dry GD column, centrifuge at 16000 g for 2mins to obtain the eluted DNA.

PCR Amplification

PCR reactions and cycling conditions (Table 1) were performed on a 96-well GenePro thermocycler (BIOER technology, England). Each reaction mixture was prepared in a volume of 20 μl, consisting of 2μl of a 1 in 100 diluted DNA extract, 6µl of distilled water, 1µl each of forward and reverse primers (100μM prepared working solution), and 10µl of Quantabio repliQa Hifi toughmix, that includes 2x reaction buffer containing optimized concentrations of $MgCl_2$, dNTP's and proprietarily formulated HiFi polymerase, hot start antibodies and ToughMix chemistry (repliQa Hifi toughmix: Quantabio, MA, USA).

Table 1 presents the PCR primers and optimal conditions. The isolates *Escherichia coli* NZRM 4396 (0178:H7, stx1 positive), *E. coli* NZRM 4397 (0171:H2, stx2 positive), *Listeria monocytogenes* NZRM 44, *Campylobacter jejuni* NZRM 2397, *Salmonella* Enterica serovar

Menston NZRM 383 and *Yersinia enterocolitica* NZRM 2603 were used to evaluate the different cycling protocols. The 16S rRNA gene (see Table 1) served as the positive control while *Pseudomonas marincola* LU P2 served as a negative control for all experiments. The specific bands of each bacterial isolate obtained under optimal conditions are presented in Appendix Figure.

Gel Electrophoresis

Each electrophoretic setup is composed of 0.8% agarose gel stained with 2µl SYBR Safe. A 0.5M TBE (Tris-borate EDTA, pH 8.0) was used as the running buffer. Each well was loaded with 2µl of PCR product after mixing with few drops of 6X 30% glycerol. An Invitrogen 1kb plus DNA ladder (Thermofisher scientific, USA) served as the molecular-weight size marker. Power was supplied to the set up at 100V for 40minutes. Electrophoresed gels were visualized using a UV-fluorescence Bio-Rad imaging system (Bio-Rad laboratories, USA).

References

1. Kawase J, Etoh Y, Ikeda T, Yamaguchi K, Watahiki M, Shima T, et al. Yamaguch i, Watahiki M, Shima T. An improved multiplex real-time SYBR Green PCR assay for the analysis of 24 target genes from 16 bacterial species in fecal DNA samples from patients with foodborne illnesses. Jpn J Infect Dis. 2016;69:191–201. PubMed https://doi.org/10.7883/yoken.JJID.2015.027

2. Linton D, Lawson AJ, Owen RJ, Stanley J. PCR detection, identification to species level, and fingerprinting of *Campylobacter jejuni* and *Campylobacter coli* direct from diarrheic samples. J Clin Microbiol. 1997;35:2568–72. PubMed https://doi.org/10.1128/jcm.35.10.2568-2572.1997

3. Chakravorty S, Helb D, Burday M, Connell N, Alland D. A detailed analysis of 16S ribosomal RNA gene segments for the diagnosis of pathogenic bacteria. J Microbiol Methods. 2007;69:330–9. PubMed https://doi.org/10.1016/j.mimet.2007.02.005

4. Waage AS, Vardund T, Lund V, Kapperud G. Detection of low numbers of *Salmonella* in environmental water, sewage and food samples by a nested polymerase chain reaction assay. J Appl Microbiol. 1999;87:418–28. PubMed https://doi.org/10.1046/j.1365-2672.1999.00835.x

5. Lantz P-G, Knutsson R, Blixt Y, Al Soud WA, Borch E, Rådström P. Detection of pathogenic *Yersinia enterocolitica* in enrichment media and pork by a multiplex PCR: a study of sample preparation and PCR-inhibitory components. Int J Food Microbiol. 1998;45:93–105. PubMed https://doi.org/10.1016/S0168-1605(98)00152-4

Appendix Table. Single specific PCR primers and optimized conditions used in the laboratory analyses

Pathogen	Gene	Primer name	Primer sequence 5′→ 3′	Product size	Cycle conditions	References
STEC	*Stx1*	Stx1-ET-F<br>stx1-ET-R	CATTACAGACTATTTCATCAGGAGGT<br>CAAATTATCCCCTGAGCCACTA	68	950C / 4 min, 950C / 10 s, 600C / 5 min, 720C / 2 s, 720C / 2 min, 100C / 1 min, 35 cycles, cycling time: 37 min	Kawase et al. (*1*)
	Stx2	stx2-ET-F<br>stx2-ET-R	CATGACAACGGACAGCAGTTAT<br>AACTCCATTAACGCCAGATATGA	114	950C / 4 min, 950C / 10 s, 600C / 5 min, 720C / 2 s, 720C / 2 min, 100C / 1 min, 35 cycles, cycling time: 37 min	Kawase et al. (*1*)
C. jejuni/coli	16S rRNA*	CCCJ609F<br>CCCJ1442R	AAT CTA ATG GCT TAA CCA TTA<br>GTA ACT AGT TTA GTA TTC CGG	854	94°C/5mins, 94°C/1min, 55°C/1min, 72°C/1min, 72°C/7mins, 10°C/1min, 25cycles, cycling time: 1h44mins	Linton et al. (*2*)
Positive control	16S rRNA	16SF<br>16SR	CCAgACTCCTACGGGAGGCAG<br>CGTATTACCGCGGCTGCTG	203	950C / 4 min, 950C / 10 s, 600C / 5 min, 720C / 2 s, 720C / 2 min, 100C / 1 min, 35 cycles, cycling time: 37 min	Chakravorty et al. (*3*)
Listeria spp	hly	Lm-hly-F<br>Lm-hly-R-kai	GGGAAATCTGTCTCAGGTGATGT<br>GTAAATTACGGCTTTGAAGGAAGA	72	950C / 4 min, 950C / 10 s, 600C / 5 min, 720C / 2 s, 720C / 2 min, 100C / 1 min, 35 cycles, cycling time: 37 min	Kawase et al. (*1*)
Salmonella spp	Nested	Sal1-F<br>Sal2-R	GTA GAA ATT CCC AGC GGG TAC TG<br>GTA TCC ATC TAG CCA ACC ATT GC	438	950C / 3 min, 950C / 30 s, 600C / 1 min, 720C / 1.5 min, 720C / 10 min, 100C / 1 min, 20 cycles, cycling time: 2 h 40 min	Waage et al. (*4*)
		Sal3-F<br>Sal4-R	TTT GCG ACT ATC AGG TTA CCG TGG<br>AGC CAA CCA TTG CTA AAT TGG CGC	312	95°C/3mins, 95°C/30secs, 67°C/1min, 72°C/2secs, 72°C/1.5mins, 10°C/1min, 40cycles, cycling time: 1h44mins	
Yersinia spp	16S rRNA**	LandzY1<br>LandzY2	GGAATTTAGCAGAGATGCTTTA<br>GGACTACGACAGACTTTATCT	300	940C / 5 min, 940C / 30 s, 580C / 30 min, 720C / 40 min, 720C / 7 min, 100C / 1 min, 30 cycles, cycling time: 1 h 21 min	Landz et al. (*5*)

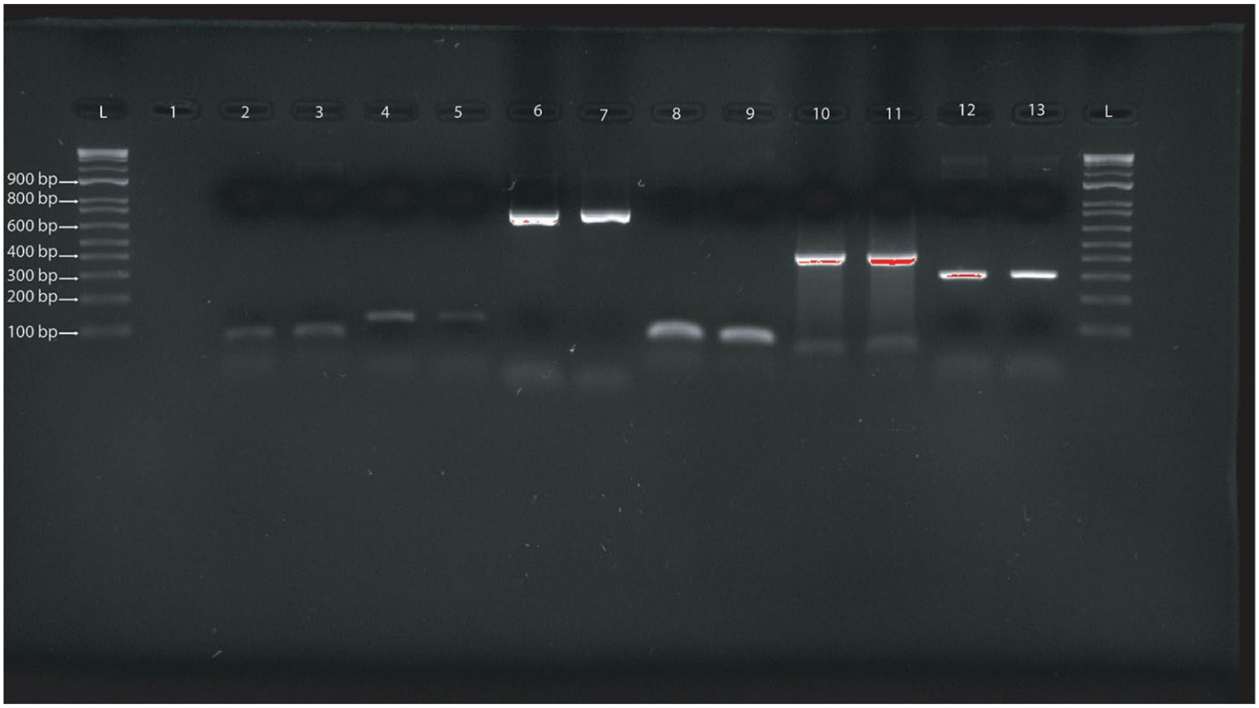

Appendix Figure. Specific bands at optimized PCR conditions. Lane L: 1kb plus DNA ladder, Lane 1: *Pseudomonas marincola* isolate (*Salmonella* spp. assay, negative control for all assays), lane 2/3: *E. coli Stx1*, lane 4/5: *E. coli Stx2* gene; lane 6/7: *Campylobacter jejuni;* lane 8/9: *Listeria monocytogenes;* lane 10/11: *Salmonella* Enterica serovar Menston; lane 12/13: *Yersinia enterocolitica.*
